# Approach to Hemoptysis in the Modern Era

**DOI:** 10.1155/2017/1565030

**Published:** 2017-12-21

**Authors:** Sébastien Gagnon, Nicholas Quigley, Hervé Dutau, Antoine Delage, Marc Fortin

**Affiliations:** ^1^Institut Universitaire de Pneumologie et de Cardiologie de Québec, 2725 Ch Ste-Foy, Quebec City, QC, Canada G1V 4G5; ^2^Hôpital Nord Marseille, Assistance Publique des Hôpitaux de Marseille, 13915 Chemin des Bourrely, Marseille, France

## Abstract

Hemoptysis is a frequent manifestation of a wide variety of diseases, with mild to life-threatening presentations. The diagnostic workup and the management of severe hemoptysis are often challenging. Advances in endoscopic techniques have led to different new therapeutic approaches. Cold saline, vasoconstrictive and antifibrinolytic agents, oxidized regenerated cellulose, biocompatible glue, laser photocoagulation, argon plasma coagulation, and endobronchial stents and valves are amongst the tools available to the bronchoscopist. In this article, we review the evidence regarding the definition, etiology, diagnostic modalities, and treatment of severe hemoptysis in the modern era with emphasis on bronchoscopic techniques.

## 1. Introduction

Hemoptysis is often an alarming and worrisome symptom for the patient and the physician. Although massive hemoptysis occurs in less than 20% of the cases [1], it can have devastating consequences. Technological advances have enabled a more effective management, especially with the introduction of bronchial artery embolization and the improvements in computed tomography and bronchoscopy. Bronchoscopy remains an important diagnostic and therapeutic procedure in hemoptysis. New techniques to control bleeding have been reported with interesting results, and older approaches have been improved. This review addresses the approach of hemoptysis in the modern era, with particular emphasis on bronchoscopy.

## 2. Definition

Hemoptysis can be of variable severity ranging from slightly blood-streaked sputum to life-threatening hemorrhage [2, 3]. Severe hemoptysis should be promptly identified as it presents a significant mortality risk which has been reported to be as high as 80% without appropriate management [4–8]. Nearly all definitions of severe or massive hemoptysis in the literature rely on the reported volume of expectorated blood in a 24-hour period. There is no consensus on a specific volume threshold for an hemoptysis to be considered massive [9, 10]. Studies use different thresholds ranging from 100 mL [11] to over 1000 mL [12]. Definitions relying on self-reported volumes are imperfect as patients may overestimate or underestimate volume as massive hemoptysis, understandably, causes significant stress [10]. We recommend to measure hemoptysis volume when patients are admitted to the hospital to have an objective assessment of clinical evolution, but unfortunately, such precise measures are seldom available on initial presentation.

## 3. Etiology

All hemoptysis etiologies can potentially cause massive hemoptysis. A broad differential diagnosis of hemoptysis is summarized in Table 1 [4, 13–27]. The most frequent causes of hemoptysis vary significantly geographically and depending on the clinical setting. Certain causes of hemoptysis have a greater tendency to be massive [18], while others are seldom massive. The five most frequent causes of massive hemoptysis worldwide, in random order, remain bronchiectasis, lung cancer, tuberculosis [4], lower respiratory tract infections, and mycetomas [16]. Their prevalence will vary from center to center because of demographic reasons as is demonstrated in Table 2 [28–37]. However, despite a thorough investigation, an important proportion of hemoptysis remains cryptogenic—up to 50%—as reported by a recent 5-year retrospective study of the French nationwide hospital database [38].

## 4. Diagnosis

The investigations of choice to diagnose the cause and localize the source of hemoptysis will vary depending on the past medical history and current presentation of a patient. Chest X-ray (CXR) remains the initial test performed in the majority of cases [39]. It determines the site of bleeding in 45 to 65% of the cases and the cause in 25 to 35% [32, 40, 41]. However, as much as 10% of pulmonary malignancies are occult on CXR, 96% of which will be detected by computed tomography (CT) [42]. For this reason, in the presence of a normal CXR, further investigation is often necessary, especially in the context of lung cancer risk factors [39]. Whether bronchoscopy or CT should be performed next has been a subject of controversy, but recent studies tend to demonstrate the superiority of CT to diagnose the cause of hemoptysis. In a prospective series including 87 patients with severe hemoptysis, Chalumeau-Lemoine et al. [43] demonstrated equivalence between flexible bronchoscopy and CT to localize the source of bleeding. However, CT was significantly better to determine the cause of hemoptysis (86% versus 70%, *p*=0.007) and resulted in a change in treatment approach in 22%. Nielsen et al. [44] showed that bronchoscopy does not add significant benefit to CT sensitivity when performed to rule out cancer after an episode of hemoptysis (0.97 versus 0.92, *p*=0.58). Bønløkke et al. [45] obtained similar results in a retrospective review of 379 patients. CT is also an important tool to help guide an embolization procedure [46–48].

During diagnostic testing for severe hemoptysis, it is important to remember that securing the patient's airway is always the priority. Performing a CT requires the patient to be transported into a setting where the clinician may be suboptimally equipped to manage a massive bleed. In certain situations, it is preferable to prophylactically secure the patient's airway prior to transportation to the radiology department, but cases need to be evaluated individually. Although CT is superior in identifying the cause of bleeding, bronchoscopy has several advantages over CT. Bronchoscopy can be performed at the bedside and does not require the patient to be transported. It can be a useful tool to help secure the airway if the bronchoscopist is experienced with pulmonary isolation techniques, and it can allow endobronchial treatments as mentioned later in this review. However, when there is active hemoptysis, bronchoscopy can stimulate coughing and therefore increase bleeding. For that reason, it is often safer to delay the bronchoscopy until initial bronchial artery embolization has been performed when the patient presents with severe hemoptysis.

## 5. Treatment

### 5.1. Bronchial Artery Embolization

Bronchial artery embolization (BAE) was first described by Rémy et al. [49] in 1974. Since then, the procedure has been improved and has become an essential tool for the management of hemoptysis. It is important to remember that most hemoptysis originate from the bronchial circulation, but in rare cases, hemoptysis may originate from the pulmonary circulation. Recent studies have shown the effectiveness of BAE in a wide array of pathologies including tuberculosis, bronchiectasis, aspergilloma, and malignancy [50–53]. Embolizing agents are numerous, but polyvinyl alcohol (usually 300–600 *µ*m) is the most frequently used, having the advantage of being nonabsorbable and available in different sizes [54, 55].

The most feared complication of BAE is embolization of the anterior spinal arteries, resulting in spinal cord ischemia. Over the last years, with the introduction of superselective catheterization, this complication is now uncommon (0-1%) [51–53].

BAE has proved its efficacy in hemoptysis of all degrees of severity, with a reported immediate clinical success rate of 82 to 98% [52, 53]. However, the longer term recurrence rate remains high, with reported values of 10 to 57% in recent studies [56, 57]. Recurrence can be secondary to incomplete embolization of all abnormal arteries, recanalization of previously embolized arteries, or recruitment of new collaterals [54]. Many factors have been associated with a higher risk of recurrence: malignant diseases [58], aspergilloma [59, 60], idiopathic bronchiectasis [57], and oozing or active bleeding on flexible bronchoscopy [1]. No data of quality assess the short- and long-term success rate of repeat embolization to our knowledge.

### 5.2. Endoscopic Treatment

The role of bronchoscopy in severe hemoptysis is not limited to a diagnostic role. Bronchoscopists may need to intervene on iatrogenic bleeding caused during a procedure since they perform procedures such as transbronchial biopsies which are associated with significant bleeding in 2.8% of the cases [61]. For noniatrogenic hemoptysis, flexible bronchoscopy is an essential procedure allowing at the same time identification of the source of bleeding, endoscopic treatment, and performance of pulmonary isolation to protect the unaffected lung. In recent years, many bronchoscopic techniques have been reported to manage significant hemorrhages with an interesting success rate [16].

For bronchoscopy to be safely performed in severe hemoptysis, an experienced bronchoscopic team and adequate equipment are needed. As previously mentioned, maintaining airway patency is a priority, requiring in some situations endotracheal intubation prior to a procedure or rigid bronchoscopy. If endotracheal intubation is performed, one must remember to use a large endotracheal tube to allow the passage of a therapeutic flexible bronchoscope which is preferred in case of hemoptysis because of its larger working channel which provides better suction. Rigid bronchoscopy allows bronchoscopists to perform local tamponade of the bleeding if the source is central and to use a wide variety of endoscopic techniques. Rigid bronchoscopy also allows selective intubation for pulmonary isolation in case of catastrophic bleeding.

#### 5.2.1. Cold Saline

In 1980, Conlan and Hurwitz [62] reported the successful use of cold saline lavage to control bleeding in 23 patients with massive hemoptysis (≥600 mL/24 h). Through a rigid bronchoscope, they performed a lavage with an average volume of 500 ml of 4°C normal saline. One patient presented sinus bradycardia and two subjects had rebleeding which required further lavage. After subsequent medical or surgical treatment, all patients were discharged free of hemoptysis. Large-volume cold saline lavages have not been studied again in more recent trials. However, smaller volume of cold saline is frequently used to control bleeding during bronchoscopy. To our knowledge, the efficacity of small-volume endobronchial cold saline to control endobronchial bleeding has never been studied.

#### 5.2.2. Vasoconstrictive Agents

The use of vasopressin analogs to control hemoptysis was first reported in 1989 by Breuer et al. [63] who compared the efficacy of intravenous and endobronchial glypressin during diagnostic bronchoscopy. The hemostatic effect was similar, but intravenous administration was associated with a plasma concentration 251 times higher than endobronchial administration, resulting in a significantly higher diastolic pressure. In 2004, Tüller et al. [64] demonstrated in a retrospective study that terlipressin or ornipressin stopped bleeding in 30 patients with persistent bleeding after 2 minutes of suctioning or with major bleeding. There was no short-term recurrence. Terlipressin was associated with a small but statistically significant impact on heart rate and blood pressure.

#### 5.2.3. Bronchoscopy-Guided Topical Hemostatic

Oxidized regenerated cellulose (ORC) is an absorbable water-insoluble derivative of cellulose recognized for its hemostatic and wound-healing properties. Valipour et al. [31] described in 2005 its use in bronchoscopy as a topical hemostatic agent in a cohort of 57 patients with massive hemoptysis and persistent bleeding despite bronchoscopic wedging, cold saline lavage, and local administration of epinephrine. A sterile ORC mesh was grasped with biopsy forceps through the working channel of a bronchoscope and then pulled back into the working channel prior to intubating the patient. The mesh was then placed selectively into the bleeding bronchus as peripherally as possible but within the endobronchial view. Immediate control of hemorrhage was achieved in 56 of 57 patients (98%). The patient with persistent bleeding underwent successful surgical treatment. The ORC mesh was absorbed in all patients, but 9% of the patients developed postobstructive pneumonia. No bleeding recurrences were observed. Two other recent smaller studies showed similar good results, without infectious complications [65, 66].

#### 5.2.4. Endobronchial Biocompatible Glue

Endobronchial *n*-butyl cyanoacrylate glue is a biocompatible product which is adhesive and solidifies on contact with humidity. Its efficacy has been evaluated in three case series to our knowledge [67–69], with interesting results. Bhattacharyya et al. [69] obtained immediate response in six patients. One patient required a repeat procedure on the day of the initial procedure. One long-term recurrence was noted after a mean follow-up of 127 ± 67 days. This patient with bronchiectasis was observed without further intervention and evolved favorably. Coiffard et al. [68] described the case of a 76-year-old woman with a metastatic pulmonary adenocarcinoma who had persistent hemoptysis despite two attempts of BAE and failure of endobronchial spigot placement. Cyanoacrylate-based glue was mixed with iodinated contrast to allow fluoroscopic guidance and follow-up imaging of the position of the glue. The immediate response rate was good, and no recurrence was observed at 48 hours. Chawla et al. [70] reported retrospectively the use of glue in 168 cases of mild or moderate hemoptysis persisting after 7 days of medical treatment or severe hemoptysis. Immediate control of hemoptysis was achieved in 151 patients (89.9%). All 17 patients without immediate control demonstrated very short-term recurrences, and they all underwent repeat glue application. Thirteen of them (7.7%) responded to a second glue application for an overall response rate of 97.6%. No recurrences were noted in a 6-month follow-up during which patients could be treated for conditions causing their hemoptysis.

#### 5.2.5. Endobronchial Stents

Endobronchial stents have been successfully used to treat hemoptysis secondary to endobronchial lesions. This technique has been described mainly as a palliative treatment in the context of advanced lung cancer. All reported cases showed good immediate outcomes [71–73]. Cases were published with silicone and covered self-expandable metal stents.

#### 5.2.6. Endobronchial Embolization Using Silicone Spigots

Endobronchial embolization using silicone spigots (EESS) was first described by Dutau et al. [74]. EESS is used to occlude segmental airways from which bleeding originates. After the bleeding segment has been identified, the spigot is grasped with biopsy forceps that are already inserted through the working channel of a flexible bronchoscope. Some authors stabilize the airway first with a rigid bronchoscope before performing flexible bronchoscopy [75]. The spigot is then positioned to occlude the segmental airway from which the bleeding originates. Multiple case reports have described this technique in a variety of conditions [75–77]. An interesting case report of successful EESS after failed BAE was published by Kho et al. [76]. The spigots were removed 4 days later, without further recurrence. A retrospective review by Bylicki et al. [75] included 9 patients and demonstrated a success rate of 78%. EESS was a temporary technique in a majority of cases, with 7 patients referred next to BAE and 2 patients referred for thoracic surgery. One patient had EESS as a definitive treatment. Spigots were removed after a median of 4 days, and 2 patients had late recurrence of hemoptysis.

#### 5.2.7. Fibrinogen-Thrombin

The injection of the prothrombotic fibrinogen-thrombin (FT) combination via flexible bronchoscopy was first reported by Tsukamoto et al. [78]. They retrospectively reported 19 cases in which thrombin was used alone. Thrombin alone was very effective in 15 cases, partially effective in one case, and ineffective in 4 cases. The FT combination was used in 14 cases, which demonstrated a very good response in 11 cases and a partial response in 3 cases. In a prospective study of 11 patients with severe hemoptysis, de Gracia et al. [79] obtained immediate control of bleeding in all patients with the injection of an FT combination. Two patients had a short-term severe hemoptysis relapse, and one had a long-term recurrence. Other case reports and small series demonstrated similar results [80, 81].

#### 5.2.8. Laser Photocoagulation

Laser photocoagulation was introduced by Dumon et al. [82] in 1982. Multiple types of medical lasers are now available. The main difference between lasers is their wavelength. With different wavelengths, the effect of the laser on the treated tissue will vary. Nd : YAG and Nd : YAP lasers, with respective wavelengths of 1060 and 1340 nm, are the most frequently used lasers in bronchoscopy as they provide a better coagulation effect. On the other hand, lasers such as CO_2_ lasers have a far greater wavelength, 10,600 nm, and will provide more of a cutting effect.

Han et al. [83] described in a retrospective review the efficacy of laser treatment for symptomatic palliation in a population of patients with central airway tumor. Out of 110 patients, 52 presented with hemoptysis. Hemoptysis completely resolved in 77% after laser treatment and partially improved in 17%. There was no procedure-related mortality. Another review of endoscopic palliative treatment of tracheobronchial tumors [84] showed a lower relatively short-term efficacy rate, with 58% of the patients being free of hemoptysis for 1 month or more following laser treatment. In other case reports, laser was also efficient in treating significant hemoptysis secondary to large central lesions [85, 86].

#### 5.2.9. Argon Plasma Coagulation

*Plasma* is a term used to describe an electrically conducting medium produced when the atoms in a gas become ionized. Argon plasma can be used to conduct an electric current from a probe inserted through the working channel of a flexible bronchoscope to an airway lesion. This electric current will then transform to thermal energy on contact with the tumor and coagulates its surface. Once coagulation of an area of the lesion is complete, the bronchial wall becomes less conductive and prevents deeper penetration of the electrical current in the airway wall which could lead to perforation [87]. Compared to laser photocoagulation during which the laser fiber needs to be aimed perpendicularly to the treated tissue, argon plasma coagulation can treat lesions that are parallel or perpendicular to the probe as the electrical current will travel through the plasma to the closest mucosal area which is the path of least resistance [88]. In a retrospective review of patients with endobronchial lesions and hemoptysis, Morice et al. demonstrated an immediate bleeding control rate of 100% with argon plasma, without recurrence on a mean follow-up period of 97 ± 92 days. Apart from its use on endobronchial tumors, argon plasma coagulation was also used in cases of hemangioma [89–91] and endobronchial endometriosis [92].

#### 5.2.10. Endobronchial Valves

Endobronchial valves were developed for endoscopic lung volume reduction in severely emphysematous patients with pulmonary hyperinflation [93]. These one-way valves allow air to escape from a pulmonary lobe but not enter it, therefore inducing atelectasis if there is no collateral ventilation. For management of massive hemoptysis, two case reports were published [94, 95] in patients with sequelae of tuberculosis who were successfully treated with endobronchial valves. This approach remains anecdotic. It is important to remember that there is a significant proportion of pneumothorax after positioning endobronchial valves [96]. This can lead to dramatic outcomes in the context of severe hemoptysis.

### 5.3. Pulmonary Isolation

**Figure 1:**
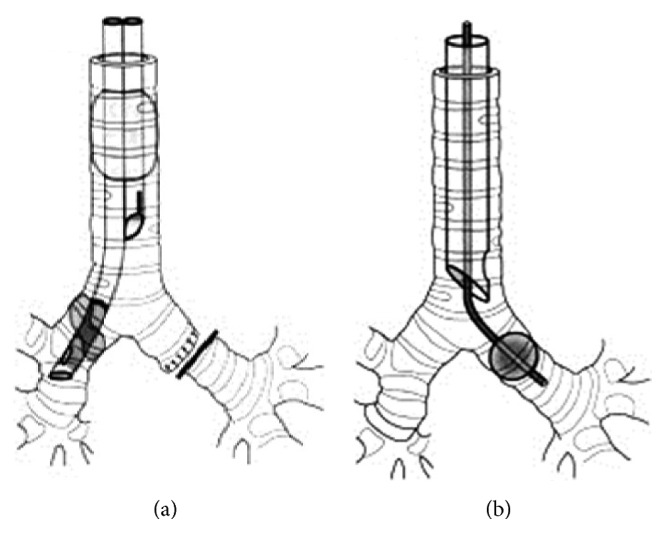
(a) Double-lumen tube. (b) Endobronchial blocker in the left mainstem bronchus.

The objective of pulmonary isolation techniques in hemoptysis is to prevent blood from the bleeding lung to enter the normal lung and, consequently, to maintain ventilation and oxygenation of the patient. A first simple maneuver is to place the bleeding source in a dependent position by turning the patient on the side of the bleeding. Trendelenburg and reverse Trendelenburg positions may also be useful depending on the position of the source of bleeding. Although elegant, positioning is not always sufficient to control blood in the bleeding lung and is not a practical solution for further management; hence, formal pulmonary isolation is occasionally needed for severe hemoptysis cases. Available pulmonary isolation techniques include selective endobronchial intubation (SEI), placement of a bronchial blocker (BB) after endotracheal intubation, and intubation with a double-lumen endotracheal tube (DLT) (Figure 1). Pulmonary isolation is not to be taken lightly, especially in an unstable patient with active severe hemoptysis. This was demonstrated by a prospective study [97] during which anesthesiologists with limited thoracic experience were asked to insert a double-lumen endotracheal tube, a Univent bronchial blocker (Fuji™, Tokyo, Japan), and an Arndt bronchial blocker (Cook Medical™, Bloomington, IN, USA) in an elective surgery setting. Twenty-two procedures were attempted with each device for a total of 66. Twenty-five (38%) attempts resulted in failures. Failure rates were similar with all the three devices. Six minutes after passage of the vocal cords with the endotracheal tube, less than half of the devices were positioned properly, while after ten minutes, more than one-third of the devices were not successfully positioned.

#### 5.3.1. Selective Endobronchial Intubation

SEI involves advancing an endotracheal tube into the mainstem bronchus contralateral to the bleeding site. This technique is generally performed under direct visualization with the bronchoscope guiding the tube into the mainstem bronchus before inflating the balloon. This technique is, in our opinion, the easiest to perform, requires no special equipment, and has the lowest cost. The main disadvantage of this technique is the risk of migration of the tube proximally. This risk is especially significant when the right mainstem bronchus, which is shorter, needs to be intubated. In this situation, the bronchoscopist must be careful not to occlude the right upper lobe.

#### 5.3.2. Bronchial Blocker

**Figure 2:**
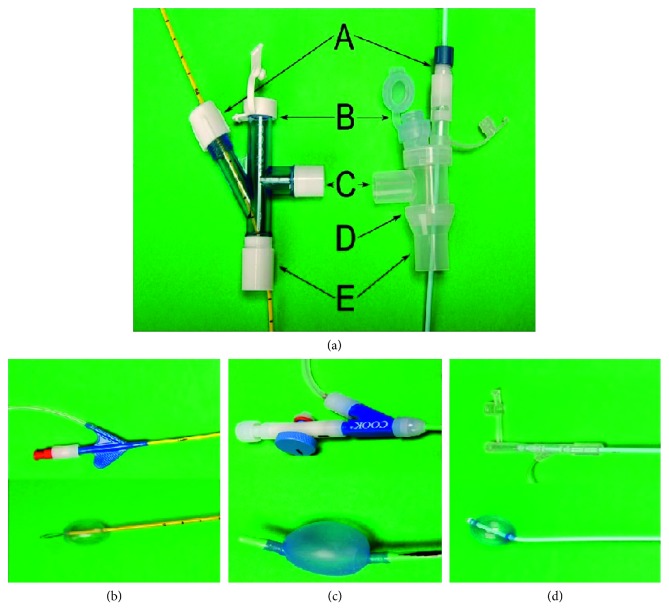
Endobronchial blockers. (a) Head of endotracheal tubes for Arndt tube on the left and Fuji blocker on the right. (A) BB port, (B) bronchoscope port, (C) ventilator port, D) Three-way connector adaptor for endotracheal tube, and (E) connector to the endotracheal tube. (b) Arndt BB (Cook Medical, Bloomington, IN, USA) with a loop at the distal end. (c) Cohen BB (Cook Medical, Bloomington, IN, USA) with a wheel to deflect the distal tip. (d) Fuji BB (Fuji, Tokyo, Japan) with a distal curve to allow positioning guidance with rotation of the proximal end.

Various devices can be passed through or beside an endotracheal tube to isolate the source of bleeding. The most frequently used devices include the Cohen tip-deflecting BB (Cook Medical, Bloomington, IN, USA), the Arndt BB (Cook Medical, Bloomington, IN, USA), and the Univent BB (Fuji, Tokyo, Japan) (Figure 2). The Cohen BB is advanced through an endotracheal tube and has a wheel to direct its tip. The Arndt BB is guided into position by passing a bronchoscope through a loop at the end of the BB and using the bronchoscope as a guide to slide the blocker in place. The Univent BB is an endotracheal tube with a channel embedded in its wall. Once the endotracheal tube is in place, the BB can be advanced into proper position under bronchoscopic guidance. Its distal tip is curved, and rotation of the proximal end of the BB allows to guide its distal end into the desired mainstem. The main advantage of the BB is that they can be used to isolate a lobe or even a segment and they simply need to be deflated to explore the bleeding source distally.

#### 5.3.3. Double-Lumen Tube

The DLT consists of two single lumens bounded together, with the longer lumen positioned in the mainstem bronchus, while a shorter lumen is positioned in the trachea to ventilate the other lung (Figure 1). The two main advantages of the DLT are that it allows ventilation of both lungs and that its positioning does not require to know the side from which the bleeding originates. The main disadvantages of the DLT include the technical difficulty in properly positioning and the small size of the two lumens which do not allow the passage of a therapeutic bronchoscope.

### 5.4. Tranexamic Acid

Tranexamic acid (TXA) is a lysine derivative that inhibits fibrinolysis through the blockage of lysine-binding sites on plasminogen [98]. Traditionally, it has been used to diminish blood loss following trauma [99], in the treatment of heavy vaginal bleeding [100] and in the perioperative management of major surgeries [101]. A limited number of trials have studied the efficacy of the administration of TXA in hemoptysis.

In 2013, Moen et al. reviewed 13 articles representing the best available evidence to address the question, “Does tranexamic acid stop hemoptysis?” [102]. Although heterogeneous in design, study population, and quality of evidence, the papers indicated that TXA may reduce both the duration and volume of hemoptysis. However, development of pulmonary embolism was described in two different case reports of patients receiving TXA. A Cochrane systematic review including two randomized controlled trials (RCTs) evaluating the effectiveness and safety of antifibrinolytic agents in hemoptysis of all causes was also published in 2012 and revised in 2016 [103]. The pooled results of the 2 RCTs from Thailand [104] (46 patients, oral administration of TXA) and Ruiz [105] (24 patients, intravenous TXA) showed a significant reduction in bleeding time in favor of the TXA group but no effect on the remission of hemoptysis at seven days.

Bellam et al. [106] conducted a randomized, controlled pilot study in 2016 comparing IV perfusion of TXA to placebo in 66 patients with submassive hemoptysis. Results showed a nonsignificant trend favoring TXA over placebo in terms of frequency and quantity of hemoptysis, need for intervention and blood transfusion, and duration of hospital stay. No adverse event was noted in the treatment arm.

While oral and intravenous routes have been the most commonly used for the administration of tranexamic acid, novel approaches have received clinical attention. In a small number of cases, aerosolized TXA and endobronchial instillation of TXA during bronchoscopy showed promising results in the treatment of hemoptysis [57, 107–111].

Even though limited research is available on the administration of tranexamic acid as a therapeutic option for hemoptysis, existing evidence appears consistent with a reduction in bleeding quantity and duration. Further clinical studies are needed, but TXA deserves consideration and may prove to be a valuable option for the treatment of severe hemoptysis. Future studies should consider that tranexamic acid has a very short half-life and, at that time, did not demonstrate effectiveness in trauma until administered as a continuous perfusion.

### 5.5. Surgery

Surgery is an effective modality to control hemoptysis in patients with localized disease [4], but patients undergoing surgery have reported mortality rates between 2% and 18% which may be a marker of severity of the underlying disease [29, 112, 113]. The mortality rate increases further when the surgery is extensive or performed in an emergency setting, reaching up to 50% [29, 114]. However, in survivors, recurrence rates are relatively low. Kiral et al. reported a 4% mortality rate with a mean follow-up of 23 months [29]. Surgery is also especially useful for conditions with a high risk of recurrence after bronchial artery embolization. For patients in whom surgery is the treatment of choice but who are not candidates on presentation, BAE can be a useful temporizing measure [115]. A multidisciplinary discussion including the surgeon, respirologist, and radiologist is often necessary in order to determine optimal management.

## 6. Conclusion

Hemoptysis remains an important and sometimes challenging medical issue. No diagnostic modality is universally superior, and each case needs to be individually approached. While bronchial artery embolization remains the cornerstone of the management of severe or persistent hemoptysis, many new endoscopic procedures have demonstrated signs of efficacy in recent years. Further data need to be obtained about the longer term results of these procedures. Comparison to placebo in addition to standard of care is also necessary, since hemoptysis frequently resolves spontaneously. Larger placebo-controlled trials of tranexamic acid perfusion in hemoptysis would also be of interest. Little is known about hemoptysis, and it remains an area of interest for future research.

## Figures and Tables

**Table 1:** Etiologies of hemoptysis.

Pulmonary-airway	Neoplasm^∗^
	Bronchitis^∗^
	Bronchiectasis^∗^
	Airway trauma
	Foreign body
	Bronchovascular fistula
Pulmonary-parenchymal	Pneumonia^∗^
	Tuberculosis^∗^
	Mycetomas^∗^(aspergillosis)/fungal infections
	Lung abcess
	Parasitic diseases
	Leptospirosis
	Cocaine inhalation
	Lung contusion
	Vasculitis (Wegener, etc.)
	Systemic lupus erythematosus
	Behcet's disease
	Goodpasture syndrome
	Idiopathic pulmonary hemosiderosis
Pulmonary-vascular	Pulmonary embolism^∗^
	Arteriovenous malformation
	Pulmonary artery pseudoaneurysm
	Dieulafoy's disease
	Pulmonary veno-occlusive disease
	Bronchial telangiectasia
Cardiac	Heart failure^∗^
	Mitral stenosis
	Congenital heart disease
Iatrogenic	Anticoagulant and antiplatelet medications
	Pulmonary artery catheter
	Biopsy
	Bronchoscopy
	Airway stent
	Endotracheal tube erosion
	Bevacizumab treatment
Others	Idiopathic^∗^
	Disorders of coagulation
	Thoracic endometriosis (catamenial hemoptysis)

^∗^The most common etiologies.

**Table 2:** Causes of massive hemoptysis in recent studies.

Study	Hemoptysis inclusion criteria	Location	*n*	Etiology of hemoptysis, %						
				TB	Bronchiectasis	Lung CA	Mycetoma	Pneumonia	Others	Idiopathic
Bhalla et al. [33]	>30 mL/d^∗^	India	64	65	9	7	NS	11	9	NS
Kiral et al. [29]	>200 mL/d	Turkey	203	21	15	20	3	NS	19	22
Fartoukh et al. [34]	ICU admission	France	1087	25	20	17	6	NS	20	18
Chan et al. [35]	>200 mL/d	Hong Kong	251	42	32	8.5	4.5	5	5	3
Shigemura et al. [36]	>600 mL/d	China	62	55	23	6	8	6	2	NS
Valipour et al. [31]	>150 mL/h	Austria	57	23	8.5	35	NS	NS	17.5	16
Ong and Eng [37]	>300 mL/d or intubation	Singapore	29	10	66	7	14	NS	3	NS
Revel et al. [32]	>150 mL/d	France	80	19	31	11	7.5	NS	21.5	10
Hsiao et al. [30]	Requiring bronchial arteriography	USA	28	7	57	14	NS	NS	14	7
Lee et al. [28]	Surgical resection considered	Hong Kong	54	17	57	4	NS	NS	7.3	NS

NS = not specified; ^∗^98% of the patients had more than 100 mL/day [33].
